# PSMA-PET/CT response after metastasis-directed radiotherapy of bone oligometastases in prostate cancer

**DOI:** 10.1186/s41824-024-00212-w

**Published:** 2024-08-19

**Authors:** Gabriel T. Sheikh, Christian Trapp, Nina-Sophie Schmidt-Hegemann, Alexander Buchner, Christian G. Stief, Marcus Unterrainer, Wolfgang G. Kunz, Clemens C. Cyran, Freba Grawe, Astrid Delker, Mathias J. Zacherl, Adrien Holzgreve, Lena M. Unterrainer, Matthias Brendel, Claus Belka, Minglun Li, Paul Rogowski

**Affiliations:** 1 Department of Nuclear Medicine, LMU University Hospital, LMU Munich, Marchioninistr. 15, 81377 Munich, Germany; 2 Department of Radiation Oncology, LMU University Hospital, LMU Munich, Munich, Germany; 3 Department of Urology, LMU University Hospital, LMU Munich, Munich, Germany; 4 Department of Radiology, LMU University Hospital, LMU Munich, Munich, Germany; 5 German Cancer Consortium (DKTK), Heidelberg, Germany; 6 German Cancer Research Center (DKFZ), Hector Cancer Institute at the University Medical Center Mannheim, Heidelberg, Germany; 7 Department of Clinical Radiology and Nuclear Medicine, Medical Faculty Mannheim, University Medical Center Mannheim, Heidelberg University, Mannheim, Germany; 8 Ahmanson Translational Theranostics Division, Department of Molecular and Medical Pharmacology, David Geffen School of Medicine, UCLA, Los Angeles, USA

**Keywords:** Oligometastatic prostate cancer (OMPC), Prostate specific membrane antigen (PSMA), PET/CT, Metastases directed therapy (MDT), Response assessment

## Abstract

**Objective:**

Bone metastases are very common in advanced prostate cancer and can sensitively be detected utilizing PSMA-PET/CT. Therefore, our goal was to evaluate the suitability of PSMA-PET/CT-guided metastasis-directed external beam radiotherapy (MDT) as treatment option for patients with biochemical recurrence and oligometastatic bone lesions.

**Materials & methods:**

We retrospectively examined 32 prostate cancer patients with biochemical recurrence and PSMA-positive oligometastatic disease limited to the bone (*n* = 1–3). A total of 49 bone lesions were treated with MDT. All patients received a post-radiotherapy PSMA-PET/CT-Scan. Changes in SUV_max_, PSMA-positive tumor volume per lesion and PSA, as well as the correlation between the PET/CT-interval and SUV_max_ response were calculated.

**Results:**

MDT lead to a SUV_max_ decrease in 46/49 (94%) of the lesions. The median relative decline of SUV_max_ was 60.4%, respectively. Based on PSMA-positive lesion volume with a SUV cut-off of 4, 46/49 (94%) of lesions showed complete response, two (4%) partial response and one lesion (2%) was stable on PSMA-PET/CT after MDT. Most of the treated patients (56.3%) showed an initial PSA decline at three months and a PSA nadir of median 0.14 ng/ml after a median time of 3.6 months after MDT. The median relative PSA change at three months after MDT was 3.9%.

**Conclusion:**

MDT is a very effective treatment modality for prostate cancer bone oligometastases and lesion response to MDT can be assessed using the (semi-)quantitative parameters SUV_max_ and PSMA-positive lesion volume with established SUV cut-offs.

## Background

Prostate cancer is the most common malignancy among men in Europe (Sung et al. 2021) and in advanced metastatic prostate cancer the incidence of bone metastases is 65–75% (Macedo et al. 2017). So far, no curative treatment option is available for prostate cancer which has metastasized to the bone. Patients with metastatic prostate cancer are currently treated with androgen deprivation therapy (ADT) and androgen receptor signaling inhibitors (ARSi) in the hormone sensitive state (mHSPC). Once the tumor becomes resistant to castration (mCRPC), therapy options include chemotherapy, radiotherapy and prostate specific membrane antigen (PSMA)-directed treatment (Cornford et al. 2017).

PSMA-positron emission tomography (PET)/computed tomography (CT) offers a high sensitivity and specificity for the detection of bone metastases (Zacho et al. 2018; Lawhn-Heath et al. 2019; Mingels et al. 2022). Furthermore, lesion identification is possible even at low serum prostate specific antigen (PSA) levels with a detection rate of 45% for PSA values between 0.20 and 0.49 ng/ml (Perera et al. 2020).

These imaging advancements combined with regular PSA-monitoring make a detection of metastatic prostate cancer at an early stage with limited metastatic count more likely (Lievens et al. 2020), a condition between localized and widespread metastatic disease termed oligometastatic disease (Hellman and Weichselbaum 1995). However, the definition of oligometastatic disease is inconsistent, with up to three or five metastases as frequent used cut-off values (Rogowski et al. 2022). The evolution of metastases is not unidirectional (Deek et al. 2021) and there is evidence that metastases can play a role in seeding further metastases (Gundem et al. 2015). On this ground, metastasis directed therapy (MDT), e.g. complete ablation by stereotactic body radiotherapy (SBRT) might improve clinical outcome. Several prospective studies have found MDT with SBRT to benefit patients with oligometastatic disease in respect to overall survival (OS) (Palma et al. 2020), progression-free survival (Tang et al. 2023), biochemical recurrence-free survival (Reyes et al. 2020), delaying biochemical or image based progression (Phillips et al. 2020) and ADT-free survival compared to other treatments or observation alone, while not being associated with any significant reduction in quality of life. However, MDT in oligometastatic prostate cancer is controversial and current national and international guidelines do not provide a clear recommendation for MDT or recommend it only within the context of studies (Cornford et al. 2021; Thomas and Schrader 2023).

Local control after MDT for bone oligometastases is high, with rates above 95% (Onal et al. 2021; Rogowski et al. 2021a, b). However, morphological assessment of the treatment response of osteoblastic bone metastases remains difficult on a lesion basis as hypersclerosis and tumor-related deformities often persist (Oprea-Lager et al. 2021). Hence, the current Response Evaluation Criteria In Solid Tumors 1.1 (RECIST1.1) guideline considers bone metastases without a significant soft tissue component to be unmeasurable and morphological imaging to be inadequate for assessing the response of bone metastases (Eisenhauer et al. 2009). PSMA-PET/CT, on the other hand, can (semi-)quantitatively assess PSMA-expression of bone metastases. Nevertheless, data investigating the treatment response of irradiated bone lesions based on repeated PET-imaging are scarce (Baumann et al. 2018).

Therefore, the aim of our study was to evaluate treatment response in patients treated with MDT for bone oligometastatic prostate cancer on pre- and post-radiotherapy PSMA-PET/CT.

## Methods

This retrospective analysis was performed in compliance with the principles of the Declaration of Helsinki and its subsequent amendments (World Medical Associations Declaration 2013) and was approved by the local Ethics Committee of the Medical Faculty (approval number 19–361).

### Patient selection

Consecutive patients undergoing MDT for bone oligorecurrent prostate cancer at the University Hospital Munich (LMU) between January 2015 and November 2022 were retrospectively identified (*n* = 32). Oligometastatic disease was defined as presence of up to three bone metastases (miM1b (oligo) according to the PROMISE v2.0 framework (Seifert et al. 2023a, b). Simultaneous intrapelvic nodal disease (miN1-2) and lymph nodes in the common iliac or retroperitoneal region (miM1a) were allowed. However, patients with distant lymph nodes in other regions, visceral metastases (miM1c) and patients with oligoprogressive or induced oligometastatic disease were excluded. All patients had hormone-sensitive prostate cancer at the time of MDT. Patients with repeated PSMA-PET/CT examinations (pre- and post-radiotherapy) were considered for analysis. The reason for performing a repeated PSMA-PET/CT investigation were persisting or increasing serum PSA levels after MDT. .

### PSMA ligand and PET/CT imaging protocol

Patients were imaged by [^68^Ga]Ga-PSMA-11- or [^18^F]PSMA-1007-PET/CT as previously described (Rogowski et al. 2021a, b). Pooling of data from different scanners (Siemens Biograph 64 and GE Discovery 690 PET/CT) was possible on the basis of phantom studies carried out by our medical physics department, resulting in a conversion factor, based on radionuclide, lesion diameter and SUV_max_. Radiolabelling was performed in conformity with good clinical practice. In absence of contraindications, patients received 20 mg furosemide at the time of tracer injection. PSMA-PET/CT scans were acquired approximately 60 min after intravenous tracer injection. Depending on previous CT scans and contraindications, a contrast-enhanced or unenhanced diagnostic CT (120 kV, 100–400 mAs, dose modulation) was used for anatomical correlation and attenuation correction.

### Image analysis

PSMA-PET/CTs were primarily interpreted in clinical routine by a junior nuclear medicine physician or radiologist and a senior nuclear medicine physician as well as a senior radiologist, the latter both with a minimum of 5 years of PET/CT experience. An independent secondary evaluation of the clinical reports and the images was carried out by another radiologist and nuclear medicine physician with 3 years of PET/CT experience. Cases of disagreement were solved in consensus. Lesion location was determined by CT. PET-positive lesions were visually identified on [^68^Ga]Ga-PSMA-11-/[^18^F]PSMA-1007-PET/CT as focal uptake above background not associated with the physiological uptake (Fendler et al. 2017). Tumor delineation and an PSMA-positive volume of bone lesions was based on a 3D isocontour at 50% of a lesions maximum SUV as recommended by the European Association of Nuclear Medicine for FDG-PET imaging (Boellaard et al. 2015) and mentioned in the PROMISE V2 Supplements for PSMA-PET/CT (Seifert et al. 2023a, b).

### Radiotherapy treatment

Treatment indications were approved by an interdisciplinary tumor board. Radiotherapy was administered to all PSMA-PET/CT positive lesions. The planning target volume (PTV) comprised the macroscopic bone lesion with a margin depending on the site and the expected intrafractional motion. All patients received volumetric modulated arc therapy (VMAT) and image-guided radiotherapy (IGRT). The exact dose prescription depended on the volume and the localization of the lesion. Patients diagnosed with local recurrence and / or pelvic lymph node-recurrence additional to bone metastases were treated simultaneously with radiotherapy to prostate fossa with or without whole-pelvic radiotherapy and boost to affected lymph nodes. The recommendation for concomitant ADT was based on disease burden, comorbidities and patient’s preference.

### Response evaluation

For the assessment of lesion response to radiotherapy SUV_max_ and PSMA-positive lesion volume of the irradiated lesions were recorded on pre- and post-radiotherapy PSMA-PET/CT-Scans and absolute and percentual changes were calculated. Based on the PSMA PET Progression framework (Fanti et al. 2020) and the consensus statements on PSMA-PET/CT response assessment criteria in prostate cancer (Fanti et al. 2021) an SUV_max_ increase of 30% was considered as progressive disease (PD), while an SUV_max_ decrease or increase below 30% was considered as non-progressive disease (non-PD).

**Fig. 1:**
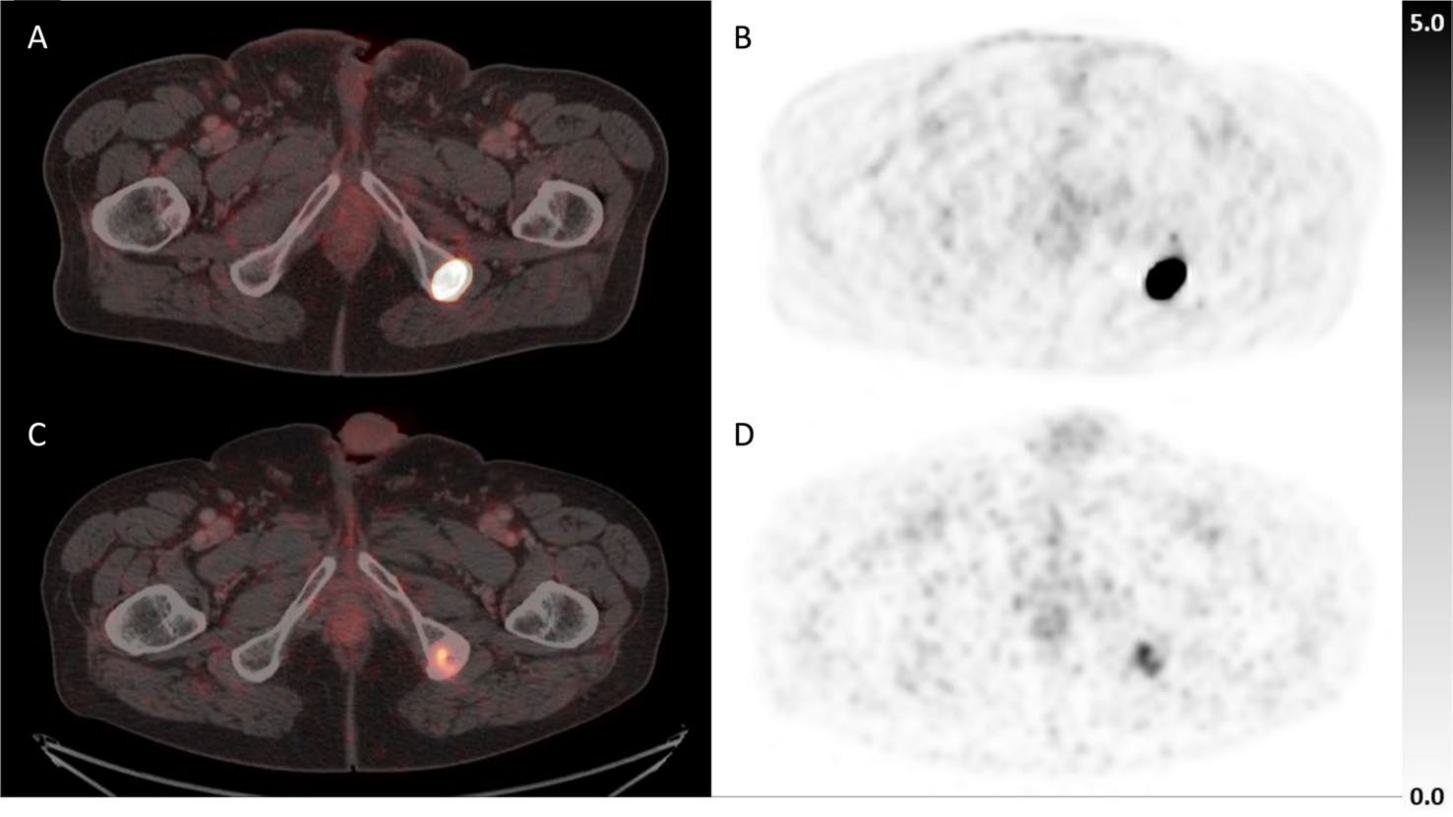
Example of bone metastasis with PR, fused PET/CT and PET only prior to (**A + B**) and after MDT (**C + D**)

**Fig. 2:**
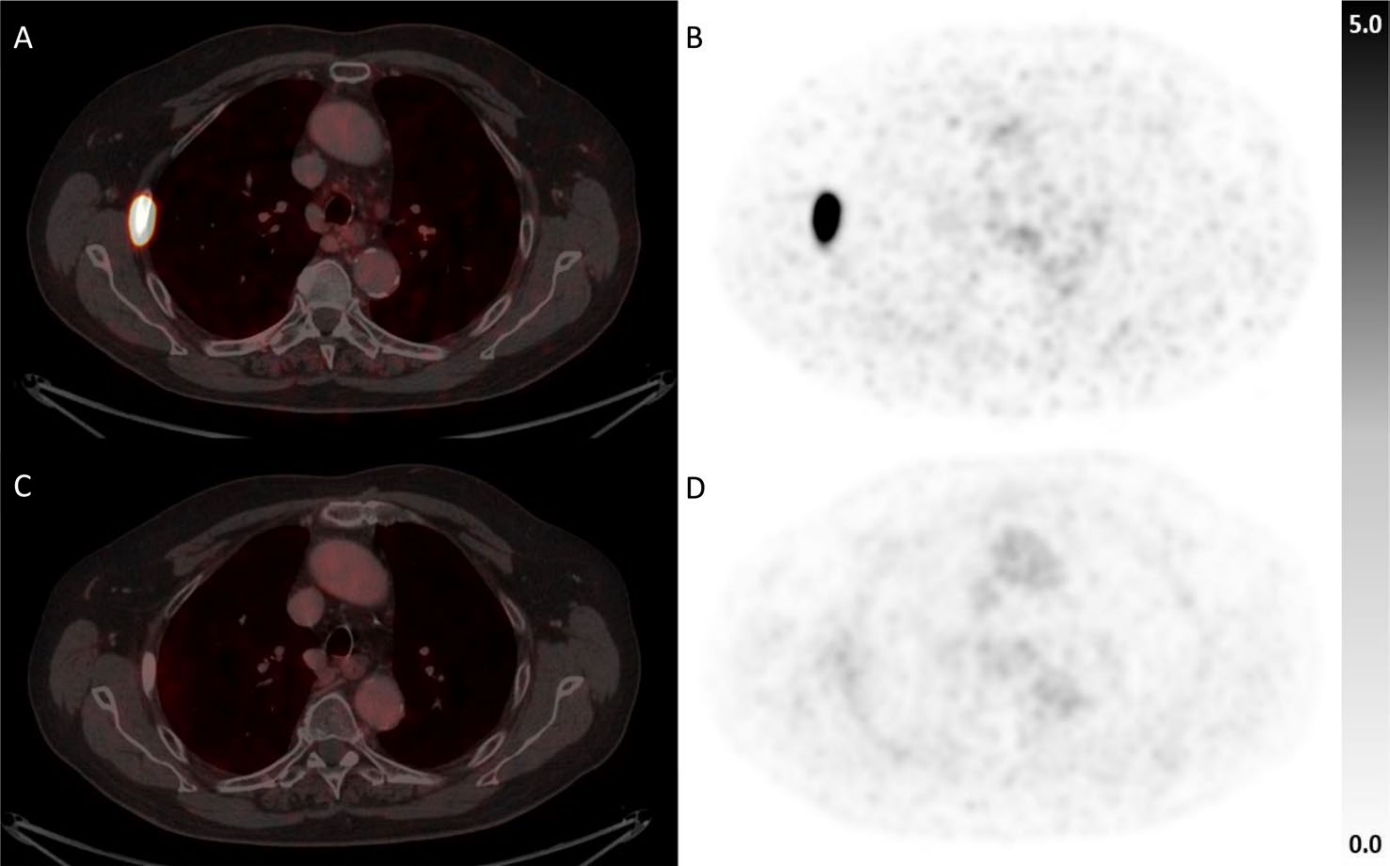
Example of bone metastasis with CR, fused PET/CT and PET only prior to (**A + B**) and after MDT (**C + D**)

Cut-offs for evaluation of PSMA-positive lesion volume were adopted from the RECIP framework (Gafita et al. 2022), with an increase in lesional tumor volume ≥ 20% confirming lesion progression, a decrease of ≥ 30% defining lesion regression (Fig. 1) and values in between defining a stable lesion. No residual PSMA-uptake above background levels on follow-up PSMA-PET/CT was considered as complete response of a lesion (Fig. 2).

Post-radiotherapy PSA-response was also evaluated. A decrease or increase in the PSA value compared to the pre-MDT level by > 0.2 ng/ml was considered as a response or progression, respectively, while values in between were considered stable.

### Statistical analysis

All statistical analyses were conducted using SPSS Version 28 (IBM Corp., Armonk, NY, USA). Descriptive statistics were used to describe patient and treatment characteristics. Continuous measures were summarized using median and range, whereas ordinal and categorial measures were summarized using counts and percentages. Mann-Whitney-U-Test was used for univariate analyses and correlation analyses were conducted using the Spearman rank test. P-values of < 0.05 were considered statistically significant.

## Results

### Demographics

A total of 32 patients treated with MDT for bone oligorecurrent disease had repeated PSMA-PET/CT examinations pre- and post-radiotherapy at the University Hospital Munich (LMU) between January 2015 and November 2022. PET/CT ligands were [68Ga]Ga-PSMA-11 and [18 F]PSMA-1007 in 28.1% and 71.9%, respectively pre- and 6.1% and 93.9%, respectively post-MDT. In six patients there was a switch from [68Ga]Ga-PSMA-11 pre-MDT to [18 F]PSMA-1007 post-MDT, three patients received [68Ga]Ga-PSMA-11 and 23 patients [18 F]PSMA-1007 pre- and post-MDT. The reason for the second PSMA-PET/CT was a PSA increase after MDT in all patients.

Patient characteristics at baseline and treatment characteristics are shown in Table 1. The median age at the time of MDT was 73.5 years (range 57–81). Primary therapy was radical prostatectomy in all patients. The initial tumor stage was T2 in 25%, T3 in 75% and N1 in 21.9%. The initial ISUP score was ≥ 4 in 71.8%. The majority of patients (62.5%) presented with a single bone metastasis in the first PSMA-PET/CT (range one to three). Seven patients (21.9%) received additional RT to the prostatic fossa and /or a pelvic RT with a boost on positive lymph nodes simultaneously with the MDT for bone metastases due to macroscopic extraosseous recurrence. The median biologically effective dose (BED) (α/β = 3) administered to bone metastases was 93.3 Gy (range 66.7–93.3 Gy). The site of treated metastases was thorax, pelvis and spine in 56.3%, 37.5% and 6.3% of patients, respectively. Eleven patients (34.4%) received concomitant ADT at a median of four days before MDT.

**Table 1:** Patient and treatment characteristics at baseline

Patients, *n*	32
Bone metastases, n	49
Age (years), median (range)	73.5 (61–86)
Initial tumor stage, n (%)	
T2	8 (25.0)
T3	24 (75.0)
Initial nodal stage, n (%)	
N0	23 (71.9)
N1	7 (21.9)
Nx	2 (6.3)
Initial ISUP score, n (%)	
2	5 (15.6)
3	4 (12.5)
4	6 (18.8)
5	17 (53.1)
Initial PSA (ng/ml), median (range)	11.4 (4-127)
Number of bone oligometastases, n (%)	
1	20 (62.5)
2	7 (21.9)
3	5 (15.6)
Site of bone oligometastases, n of lesions (%)	
Thorax	18 (56.3)
Spine	2 (6.3)
Pelvis	12 (37.5)
RT fractionation, n of lesions (%)	
30 Gy, 5 fractions (BED_3_ = 90.0 Gy)	18 (36.7)
40 Gy, 10 fractions (BED_3_ = 93.3 Gy)	24 (49.0)
other (BED_3_ 66.7–93.3 Gy)	7 (14.3)
Concomitant ADT, n (%)	
yes	11 (34.3)
no	21 (65.6)

*Abbreviations* ADT = androgen deprivation therapy; BED_3_ = biologically effective dose, α/β = 3; ISUP = International Society of Urological Pathology; PSA = prostate specific antigen; RT = radiotherapy

### PSMA-PET/CT response

**Fig. 3:**
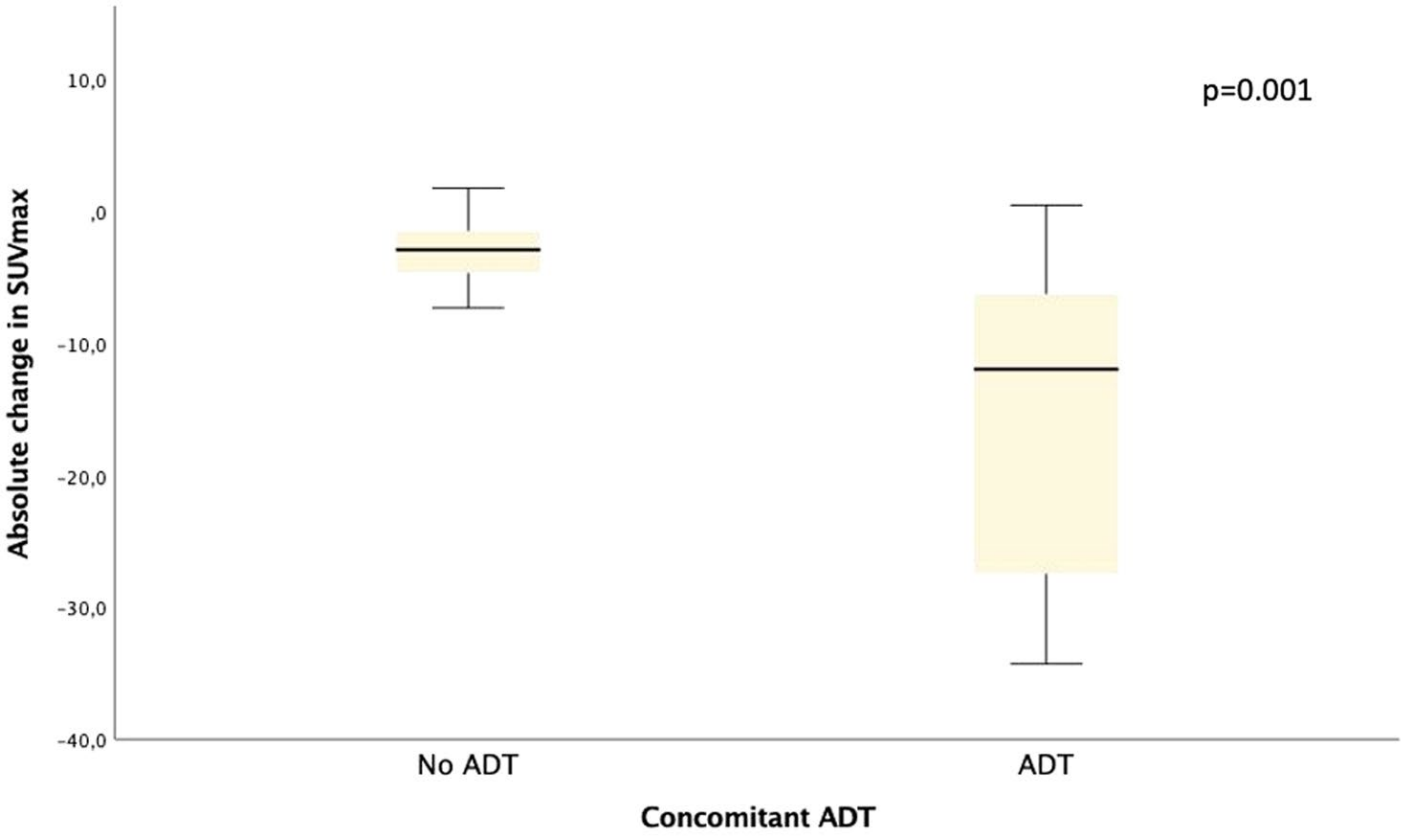
Absolute change in SUV_max_ in patients with and without concomitant ADT to radiotherapy

The median interval between the first and the second PSMA-PET/CT was 13.2 months (range 4.4–63.3 months). The median SUV_max_ was 5.19 (range 2.3–87.4) and 1.93 (range 0,67–5.78) on pre- and post-MDT PET/CT, respectively. The median absolute and relative change of SUV_max_ was − 2.97 (range − 82.05 to + 1.76) and − 60.4% (range − 98% to + 54%), respectively. The median absolute change of SUV_max_ in patients with and without concomitant ADT was − 12.0 (range − 82.05 to + 0.44) and − 2.92 (range − 7.32 to + 1.76), respectively (*p* = 0.001) (Fig. 3). Based on SUV_max_ 47/49 lesions (96%) were classified as non-progressive, of which 46 lesions had any decrease of SUV_max_ and one lesion showed an increase of only 16%. Two lesions (4%) showed an increase of SUV_max_ after MDT of 54% and 30%, respectively, and were therefore classified as non-responding lesions (non-responding lesion 1 and 2, respectively).

The median PSMA-positive lesion volume was 0.66 cm3 (range 0.2–9.4 cm3) and 0.0 cm3 (range 0.0–0.93 cm3) on pre- and post-MDT PET/CT, respectively. Based on PSMA-positive lesion volume 40/49 lesions (82%) did not have any correlate above background on post-MDT PET/CT, consistent with complete response. Seven patients showed partial response with volume decreases reaching from − 66% to -95% in one lesion with a decrease of 16% was labelled as stable. When applying an SUV cut-off of 4, previously reported to be best suited to delineate the true tumor volume on [18 F]PSMA-1007-PET/CT (Mittlmeier et al. 2021), complete response was seen in 46/49 lesions (94%).

Non-responding lesion 1, which was the same lesion that was classified as stable based on PSMA-positive lesion volume, showed delayed complete response in a subsequent [18 F]PSMA-1007-PET/CT 15 months after treatment. Unfortunately there was no subsequent PSMA-PET/CT available for non-responding lesion 2, which was classified as partially responsive based on PSMA-positive lesion volume with a decrease of 94%.

**Fig. 4:**
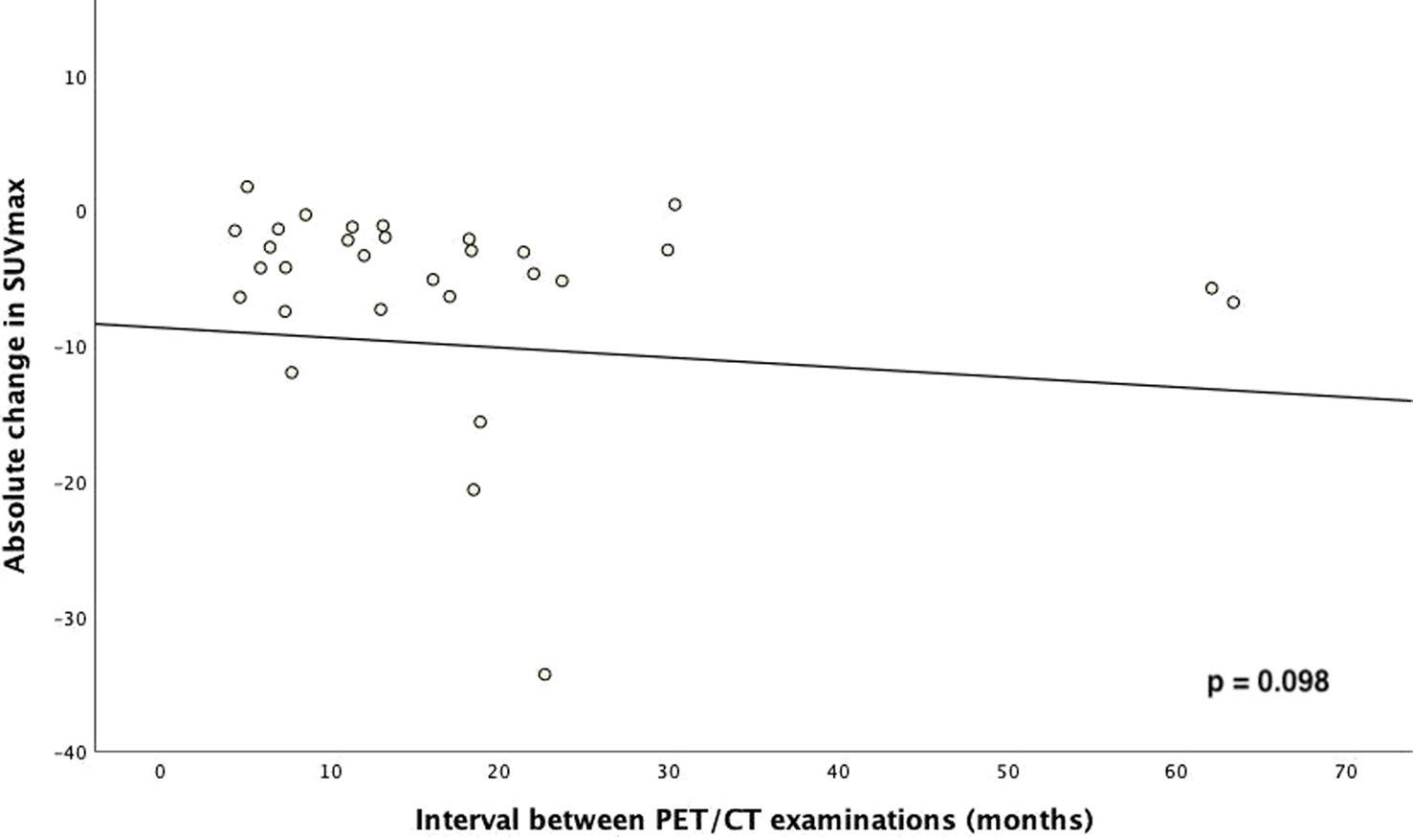
Correlation between absolute change in SUV_max_ and interval between PET/CT examinations

Twenty-one patients (65.6%) showed new metastases on post-treatment scan. In seven patients (21.9%) no suspicious lesion could be found on PSMA-PET/CT, despite of biochemical recurrence. The rest of the patients (12.5%) had progressive lesions only apparent in hindsight (*n* = 2) or local recurrence of a previously treated lesion. There was no significant correlation between the interval between the two PET/CT and the SUV_max_ response (*r* = 0.282, *p* = 0.098, Fig. 4).

### PSA response

The median serum PSA values at the time of the pre- and post-radiotherapy PSMA-PET/CT were 1.03 ng/ml (range 0.2–39.2 ng/ml) and 1.05 ng/ml (range 0.1–10.3 ng/ml), respectively. The majority of treated patients (56.3%) showed an initial PSA decline three months after MDT and a median PSA nadir of 0.14 ng/ml (range 0.01–2.75 ng/ml) after a median time of 3.6 months (range 1.2–24.4 months) after MDT. However, after a median time of 15.7 months, all patients with an initial decline of PSA had a PSA-progression. The PSA was stable or rising at the first follow-up at three months in the remaining 14 patients. The median absolute and relative PSA change at three months after MDT was − 0.2 ng/ml (range − 22.9 ng/ml to + 2.48ng/ml) and − 3.9% (range − 99.9% to + 224.0%). Four patients had rising PSA values after MDT while on ADT, meeting the criteria of mCRPC.

## Discussion

Bone metastases are the most frequent site of distant metastasis in prostate cancer and PSMA-PET/CT is evolving as the standard of care imaging method for metastatic prostate cancer (Zacho et al. 2018; Gandaglia et al. 2014). Several prospective trials have demonstrated that MDT in prostate cancer may prolong the initiation of systemic therapy and the time until disease progression (Tang et al. 2023; Reyes et al. 2020; Phillips et al. 2020). However, MDT is subject to critical evaluation because its impact on overall survival is still pending in trials with prostate cancer as the sole histology (Palma et al. 2020).

Current response criteria like RECIST 1.1 and PERSIST either deem bone lesions without significant soft tissue component unmeasurable or are not applicable to imaging with PSMA-PET/CT (Eisenhauer et al. 2009). We therefore aimed to investigate treatment response of MDT for bone oligometastatic disease in prostate cancer patients based on the objective parameters SUV_max_ and PSMA-positive lesion volume on pre- and post-MDT imaging with PSMA-PET/CT.

Two different PET/CT ligands were used for imaging. In January 2018 our nuclear medicine department replaced [^68^Ga]Ga-PSMA-11 with [^18^F]PSMA-1007 in clinical routine. Even though [^68^Ga]Ga-PSMA-11 has been prospectively validated to have excellent sensitivity, especially for bone metastases (Lawhn-Heath et al. 2019), imaging with ^18^F has several advantages over ^68^Ga. The longer half-life and the possibility of large batch production make handling easier, while the lower positron energy of ^18^F in theory increases the resolution (Kesch et al. 2017). A potential pitfall of [^18^F]PSMA-1007 on the other hand is the increased frequency of unspecific focal bone uptake (UBU) that has previously been reported (Grünig et al. 2021; Seifert et al. 2023a, b), which can lead to a decreased diagnostic accuracy for bone lesions (Mingels et al. 2022) and inadequate therapy. Unfortunately, there is no definitive way of differentiating between UBU and a true lesion in the retrospective setting and a standardized definition of UBU is lacking (Seifert et al. 2023a, b; Grünig et al. 2021; Phelps et al. 2023). It is therefore possible that some of the lesions we evaluated did not represent prostate cancer metastases.

We used a SUV_max_ increase above 30% to define a non-responding lesion, adopted from the PSMA PET Progression criteria (PPP) and the consensus statements on PSMA-PET/CT response assessment criteria in prostate cancer (Fanti et al. 2020, 2021) as well as volume cut-offs defined in the RECIP framework (Gafita et al. 2022) to evaluate lesion response to MDT on PMSA-PET/CT. The PPP framework by Fanti et al. defines treatment response using three different criteria, one of which is the increase in PSMA uptake of one or more existing lesions by at least 30% (Fanti et al. 2020). The RECIP framework by Gafita et al. takes into account the total PSMA-positive tumor volume and the presence or absence of new lesions (Gafita et al. 2022). Both frameworks evaluate tumor response and progression on patient basis and could therefore only be used in parts for our lesion based response assessment. While Gafita et al. propose a SUV cut-off of 3 to define PSMA-positive tumor volume on [^68^Ga]Ga-PSMA-11, Mittlmeier et. described a SUV cut-off of 4 to be best suited to delineate PSMA-positive tumor volume on [^18^F]PSMA-1007-PET/CT. Since some of the lesions treated with MDT were very small and already had a SUV_max_ below 3 on pre-MDT PET/CT, we utilized a relative threshold of 50% of local SUV_max_ which can be superior to fixed thresholds due to partial volume effects as also stated in the supplements of the second version of the prostate cancer molecular imaging standardized evaluation framework including response evaluation for clinical trials (PROMISE V2 (Seifert et al. 2023a, b) based on the work by Erdi et al. (1995, 1997). Morphologic imaging was not helpful in response assessment, as many lesions lacked well delineated morphologic correlates on pre- as well as post therapeutic PSMA-PET/CT. Our analysis of PET response after MDT of bone metastases showed very high response rates up to 96% based on SUV_max_ as well as PSMA-positive lesion volume, well in line with local control rates - usually defined as absence of morphological or metabolic progression - between 95% and 98% reported in literature (Onal et al. 2021; Rogowski et al. 2021a, b; Henkenberens et al. 2020). In our study the high response rate was achieved despite of a comparatively low BED_3_ of median 93.3 Gy. Other studies have found that BED-values > 100 Gy and > 108 Gy, respectively, are associated with improved outcome (Ost et al. 2016; Hurmuz et al. 2020). Only two lesions showed a significant SUV_max_ progression despite of MDT. However, the interval between the pre- and post-therapeutic PSMA-PET/CT was less than six months in both cases. Baumann et al. reported a correlation between the time interval after radiotherapy and PET-response suggesting that an interval of six months or more may be required to fully estimate the efficacy of radiotherapy in PSMA-PET imaging (Baumann et al. 2018). As a matter of fact, one of the patients showed a complete response of the treated lesion on repeat PSMA-PET 15 months after MDT. The other patient could not be evaluated in this regard due to the lack of additional follow-up PET scans. We could not confirm a correlation between the time interval and the SUV_max_ response, probably due to a long median interval of 13 months with a range of four to 63 months in our study.

SUV_max_ response was higher in patients with ADT concomitant to MDT. This might indicate a synergistic effect of radiotherapy and ADT as has been postulated before (Locke et al. 2015; Anderson and McBride 2022). This is interesting as MDT for oligometastatic disease is often investigated with the goal to defer systemic therapy (Ost et al. 2018). An increased PSMA-expression, in particular of bone metastases, has been described in patients receiving ADT and therefore could also be responsible for the difference in SUV_max_ response compared to patients not on ADT during MDT (Malaspina et al. 2023). However, the influence of ADT on PSMA-expression is complex, depending on the duration and type of ADT, and there is heterogeneity in the literature regarding the effects of ADT on PSMA expression, with some studies reporting increased PSMA uptake and others observing a decrease, particularly with long-term ADT (Vaz et al. 2020).

All patients in our study had a selection bias, since a persistent or rising PSA value after MDT was the indication for repeated PET imaging. Two thirds of the patients revealed new lesions on the second PET/CT. The informative value of the PSA response in our patient collective is thus limited by the fact that these new lesions may have already contributed to the serum PSA at the first follow-up 3 months after MDT. This underlines the importance of repeated imaging, which enables a lesion-based assessment, whereas the PSA value only provides global information about the state of the metastatic disease. Progression of some lesions may therefore be masked by the response of other lesions when looking at PSA values only (Kuten et al. 2019). Despite of this bias, most of our patients showed an initial drop of PSA levels at the first follow-up, indicating that PSMA-PET/CT-based MDT in oligometastatic prostate cancer is able to temporarily reduce the main tumor burden in the majority of patients. However, after a median time of 15.7 months all patients with an initial decline of PSA showed an increase of PSA-levels, which also has to be seen in the context of the abovementioned selection bias. In seven patients (21.9%) no suspicious lesion was found on PSMA-PET/CT at the time of biochemical recurrence after MDT (median PSA-level of 1.05 ng/ml), which matches the previously reported sensitivity of PSMA-PET/CT for biochemical recurrence (Hofman et al. 2018).

Our study has several limitations. The limited number of patients and the lack of statistical design or power make it difficult to draw a robust conclusion. Our study also has an observational nature and had no pre-defined endpoint, making it more vulnerable to bias. The different PSMA-compounds and scanners used as well as loss to follow up might also have a significant impact on the results. Furthermore, because histologic verification was not performed, we could not exclude false-positive and false-negative PSMA-PET lesions. However, sensitivity and specificity for detecting bone metastases are high (Zacho et al. 2020). Moreover, concomitant ADT was inconsistently administered, which complicated the interpretation of PSA kinetics. Nevertheless, we believe that our study adds important information to the sparse data regarding response evaluation of MDT for bone metastases of prostate cancer based on repeated PSMA-PET-imaging.

## Conclusion

The ability to assess response at the lesion level is particularly important in oligometastatic prostate cancer patients treated with MDT. Serum PSA levels only provide global information on tumor burden and are therefore not suitable for response assessment in this setting, as responding lesions could compensate for progressive or even new lesions. With PSMA-PET/CT, on the other hand, it is possible to reliably assess tumor load on a lesion basis. Using SUV_max_ and PSMA-positive lesion volume, we were able to confirm an excellent response of bone oligometastases to MDT, with almost all treated lesions showing a significant if not complete response.

## Data Availability

Research data are stored in an institutional repository and will be shared upon request to the corresponding author.

## Abbreviations

ADT: Androgen deprivation therapy
BED: Biologically effective dose
CT: Computed tomography
IGRT: Image-guided radiotherapy
MDT: Metastasis-directed therapy
mCRPC: Metastatic castration resistant prostate cancer
mHSPC: Metastatic hormone sensitive prostate cancer
OS: Overall survival
PFS: Progression-free survival
PTV: Planning target volume
PSA: Prostate-specific antigen
PSMA-PET/CT: Prostate-specific membrane antigen positron emission tomography / computed tomography
RECIST1.1: Response Evaluation Criteria In Solid Tumors 1.1
RT: Radiotherapy
SBRT: Stereotactic body radiotherapy
SIB: Simultaneous integrated boost
SUV: Standardized uptake value
VMAT: Volumetric modulated arc therapy

## References

[CR45] Anderson EM, McBride SM (2022) The Use of Androgen Deprivation Therapy in Combination with Radiation for localized prostate Cancer. Front Urol 2:890814 10.3389/fruro.2022.890814

[CR23] Baumann R et al (2018) Oligometastases in prostate cancer: metabolic response in follow-up PSMA-PET-CTs after hypofractionated IGRT. Strahlenther Onkol 194(4):318–324 29181556 10.1007/s00066-017-1239-1 PMC5869895

[CR28] Boellaard R et al (2015) FDG PET/CT: EANM procedure guidelines for tumour imaging: version 2.0. Eur J Nucl Med Mol Imaging 42(2):328–354 25452219 10.1007/s00259-014-2961-x PMC4315529

[CR3] Cornford P et al (2017) EAU-ESTRO-SIOG guidelines on prostate Cancer. Part II: treatment of relapsing, metastatic, and castration-resistant prostate Cancer. Eur Urol 71(4):630–642 27591931 10.1016/j.eururo.2016.08.002

[CR17] Cornford P et al (2021) EAU-EANM-ESTRO-ESUR-SIOG guidelines on prostate cancer. Part II—2020 update: treatment of relapsing and metastatic prostate cancer. Eur Urol 79(2):263–282 33039206 10.1016/j.eururo.2020.09.046

[CR11] Deek MP, Phillips RM, Tran PT (2021) Local therapies in Oligometastatic and oligoprogressive prostate Cancer. Semin Radiat Oncol 31(3):242–249 34090651 10.1016/j.semradonc.2021.03.007 PMC8189311

[CR22] Eisenhauer EA et al (2009) New response evaluation criteria in solid tumours: revised RECIST guideline (version 1.1). Eur J Cancer 45(2):228–247 19097774 10.1016/j.ejca.2008.10.026

[CR39] Erdi YE et al (1995) Threshold estimation in single photon emission computed tomography and planar imaging for clinical radioimmunotherapy. Cancer Res 55(23Supplement):5823s–5826s 7493353

[CR40] Erdi YE et al (1997) Segmentation of lung lesion volume by adaptive positron emission tomography image thresholding. Cancer: Interdisciplinary Int J Am Cancer Soc 80(S12):2505–2509 10.1002/(SICI)1097-0142(19971215)80:12+<2505::AID-CNCR24>3.0.CO;2-F 9406703

[CR29] Fanti S, Hadaschik B, Herrmann K (2020) Proposal for systemic-therapy response-assessment criteria at the time of PSMA PET/CT imaging: the PSMA PET progression criteria. Soc Nuclear Med. pp. 678–682 10.2967/jnumed.119.233817 PMC7198387 31806774

[CR30] Fanti S et al (2021) Consensus statements on PSMA PET/CT response assessment criteria in prostate cancer. Eur J Nucl Med Mol Imaging 48:469–476 32617640 10.1007/s00259-020-04934-4 PMC7835167

[CR27] Fendler WP et al (2017) 68Ga-PSMA PET/CT: Joint EANM and SNMMI procedure guideline for prostate cancer imaging: version 1.0. Eur J Nucl Med Mol Imaging 44(6):1014–1024 28283702 10.1007/s00259-017-3670-z

[CR31] Gafita A et al (2022) Novel Framework for treatment response evaluation using PSMA PET/CT in patients with metastatic castration-resistant prostate Cancer (RECIP 1.0): an International Multicenter Study. J Nucl Med 63(11):1651–1658 35422442 10.2967/jnumed.121.263072 PMC9635677

[CR33] Gandaglia G et al (2014) Distribution of metastatic sites in patients with prostate cancer: a population-based analysis. Prostate 74(2):210–216 24132735 10.1002/pros.22742

[CR35] Grünig H et al (2021) Focal unspecific bone uptake on [18F]-PSMA-1007 PET: a multicenter retrospective evaluation of the distribution, frequency, and quantitative parameters of a potential pitfall in prostate cancer imaging. Eur J Nucl Med Mol Imaging 48(13):4483–4494 34120201 10.1007/s00259-021-05424-x PMC8566387

[CR12] Gundem G et al (2015) The evolutionary history of lethal metastatic prostate cancer. Nature 520(7547):353–357 25830880 10.1038/nature14347 PMC4413032

[CR9] Hellman S, Weichselbaum RR (1995) Oligometastases. J Clin Oncol 13(1):8–10. 10.1200/JCO.1995.13.1.8 7799047

[CR41] Henkenberens C et al (2020) Efficacy of repeated PSMA PET-directed radiotherapy for oligorecurrent prostate cancer after initial curative therapy. Strahlenther Onkol 196(11):1006–1017 32399639 10.1007/s00066-020-01629-5 PMC7581615

[CR50] Hofman MS et al (2018) Prostate-specific membrane antigen PET: clinical utility in prostate cancer, normal patterns, pearls, and pitfalls. Radiographics 38(1):200–217 29320333 10.1148/rg.2018170108

[CR43] Hurmuz P et al (2020) Treatment outcomes of metastasis-directed treatment using 68Ga-PSMA-PET/CT for oligometastatic or oligorecurrent prostate cancer: Turkish Society for Radiation Oncology group study (TROD 09 – 002). Strahlentherapie und Onkologie, pp. 1034–1043 10.1007/s00066-020-01660-6 32617620

[CR34] Kesch C et al (2017) 68Ga or 18F for prostate cancer imaging? J Nucl Med 58(5):687–688 28408526 10.2967/jnumed.117.190157

[CR49] Kuten J et al (2019) [68Ga] Ga-PSMA-11 PET/CT for monitoring response to treatment in metastatic prostate cancer: is there any added value over standard follow-up? EJNMMI Res 9(1):1–8 31468235 10.1186/s13550-019-0554-1 PMC6715755

[CR5] Lawhn-Heath C et al (2019) Single-center prospective evaluation of 68Ga-PSMA-11 PET in biochemical recurrence of prostate cancer. Am J Roentgenol 213(2):266–274 31039025 10.2214/AJR.18.20699

[CR8] Lievens Y et al (2020) Defining oligometastatic disease from a radiation oncology perspective: an ESTRO-ASTRO consensus document. Radiotherapy Oncology: J Eur Soc Therapeutic Radiol Oncol 148:157–166 10.1016/j.radonc.2020.04.003 32388150

[CR44] Locke JA et al (2015) Synergistic action of image-guided radiotherapy and androgen deprivation therapy. Nat Reviews Urol 12(4):193–204 10.1038/nrurol.2015.50 25800395

[CR2] Macedo F et al (2017) Bone metastases: an overview. Oncol Reviews, 11(1) 10.4081/oncol.2017.321 PMC5444408 28584570

[CR47] Malaspina S et al (2023) Flare on [18F] PSMA-1007 PET/CT after short-term androgen deprivation therapy and its correlation to FDG uptake: possible marker of tumor aggressiveness in treatment-naïve metastatic prostate cancer patients. Eur J Nucl Med Mol Imaging 50(2):613–621 36161511 10.1007/s00259-022-05970-y PMC9816233

[CR6] Mingels C et al (2022) Diagnostic accuracy of [18F] PSMA-1007 PET/CT in biochemical recurrence of prostate cancer. Eur J Nucl Med Mol Imaging 49(7):2436–2444 35067735 10.1007/s00259-022-05693-0 PMC9165245

[CR32] Mittlmeier LM et al (2021) Feasibility of different tumor delineation approaches for 18F-PSMA-1007 PET/CT imaging in prostate cancer patients. Front Oncol, : p. 1612 10.3389/fonc.2021.663631 PMC8176856 34094956

[CR19] Onal C et al (2021) Oligometastatic bone disease in castration-sensitive prostate Cancer patients treated with stereotactic body Radiotherapy using 68Ga-PSMA PET/CT: TROD 09 – 004 study. Clin Nucl Med 46(6):465–470 33661210 10.1097/RLU.0000000000003558

[CR21] Oprea-Lager DE et al (2021) Bone metastases are measurable: the role of whole-body MRI and Positron Emission Tomography. Front Oncol 11(November):1–18 10.3389/fonc.2021.772530 PMC8640187 34869009

[CR42] Ost P et al (2016) Progression-free Survival following stereotactic body radiotherapy for oligometastatic prostate Cancer Treatment-naive recurrence: a multi-institutional analysis. Eur Urol 69(1):9–12 26189689 10.1016/j.eururo.2015.07.004

[CR46] Ost P et al (2018) Surveillance or metastasis-directed therapy for oligometastatic prostate cancer recurrence: a prospective, randomized, multicenter phase II trial. J Clin Oncol 36(5):446–453 29240541 10.1200/JCO.2017.75.4853

[CR13] Palma DA et al (2020) Stereotactic ablative radiotherapy for the comprehensive treatment of oligometastatic cancers: long-term results of the SABR-COMET phase II randomized trial. J Clin Oncol 38(25):2830–2838 32484754 10.1200/JCO.20.00818 PMC7460150

[CR7] Perera M et al (2020) Gallium-68 prostate-specific membrane Antigen Positron Emission Tomography in Advanced prostate Cancer—updated diagnostic utility, sensitivity, specificity, and distribution of prostate-specific membrane Antigen-avid lesions: a systematic review and Meta. Eur Urol 77(4):403–417 30773328 10.1016/j.eururo.2019.01.049

[CR38] Phelps TE et al (2023) Predicting outcomes of Indeterminate Bone lesions on 18F-DCFPyL PSMA PET/CT scans in the setting of high-risk primary or recurrent prostate Cancer. J Nucl Med 64(3):395–401 36265908 10.2967/jnumed.122.264334 PMC11927076

[CR16] Phillips R et al (2020) Outcomes of Observation vs Stereotactic Ablative Radiation for oligometastatic prostate Cancer: the ORIOLE phase 2 Randomized Clinical Trial. JAMA Oncol 6(5):650–659 32215577 10.1001/jamaoncol.2020.0147 PMC7225913

[CR15] Reyes DK et al (2020) Multidisciplinary total eradication therapy (TET) in men with newly diagnosed oligometastatic prostate cancer. Med Oncol 37(7):1–12 10.1007/s12032-020-01385-7 PMC7286864 32524295

[CR20] Rogowski P et al (2021a) Outcomes of metastasis-directed therapy of bone oligometastatic prostate cancer. Radiat Oncol 16(1):1–11 34193194 10.1186/s13014-021-01849-8 PMC8247211

[CR26] Rogowski P et al (2021b) Outcome after PSMA-PET/CT-based salvage radiotherapy for nodal recurrence after radical prostatectomy. European Journal of Nuclear Medicine and Molecular Imaging 10.1007/s00259-021-05557-z PMC8921036 34628521

[CR10] Rogowski P et al (2022) Radiotherapy in oligometastatic prostate cancer—a pattern of care survey among members of the German Society for Radiation Oncology (DEGRO). Strahlenther Onkol 198(8):727–734 35364690 10.1007/s00066-022-01925-2 PMC9300519

[CR25] Seifert R et al (2023a) Second version of the prostate cancer molecular imaging standardized evaluation framework including response evaluation for clinical trials (PROMISE V2). Eur Urol 10.1016/j.eururo.2023.02.002 36935345

[CR36] Seifert R et al (2023b) Unspecific 18F-PSMA-1007 bone uptake evaluated through PSMA-11 PET, bone scanning, and MRI triple validation in patients with biochemical recurrence of prostate cancer. J Nucl Med 64(5):738–743 36460340 10.2967/jnumed.118.215434

[CR1] Sung H et al (2021) Global Cancer statistics 2020: GLOBOCAN estimates of incidence and Mortality Worldwide for 36 cancers in 185 countries. Cancer J Clin 71(3):209–249 10.3322/caac.21660 33538338

[CR14] Tang C et al (2023) Addition of Metastasis-Directed therapy to intermittent hormone therapy for oligometastatic prostate Cancer: the EXTEND phase 2 Randomized Clinical Trial. JAMA Oncol 10.1001/jamaoncol.2023.0161 PMC10080407 37022702

[CR18] Thomas C, Schrader A (2023) Neue S3-Leitlinie Prostatakarzinom 2021 (Version 6.2)–Was hat sich beim fortgeschrittenen Prostatakarzinom geändert? Die Urologie 62(2):171–175 36066611 10.1007/s00120-022-01927-z PMC9911494

[CR48] Vaz S et al (2020) Influence of androgen deprivation therapy on PSMA expression and PSMA-ligand PET imaging of prostate cancer patients. Springer, pp 9–15 10.1007/s00259-019-04529-8 31654093

[CR24] World Medical Association Declaration of Helsinki: ethical principles for medical research involving human subjects. JAMA, (2013) 310(20): p. 2191–2194 10.1001/jama.2013.281053 24141714

[CR4] Zacho HD et al (2018) 68Ga-PSMA PET/CT for the detection of bone metastases in prostate cancer: a systematic review of the published literature. Clin Physiol Funct Imaging 38(6):911–922 10.1111/cpf.12480 29082604

[CR51] Zacho HD et al (2020) Added value of 68Ga-PSMA PET/CT for the detection of bone metastases in patients with newly diagnosed prostate cancer and a previous 99mTc bone scintigraphy. EJNMMI Res, 10(1) 10.1186/s13550-020-00618-0 PMC7142208 32270300

